# A Scoping Review of Hyaluronidase Use in Managing the Complications of Aesthetic Interventions

**DOI:** 10.1007/s00266-022-03207-9

**Published:** 2022-12-19

**Authors:** Ali Borzabadi-Farahani, Afshin Mosahebi, David Zargaran

**Affiliations:** 1 Division of Surgery & Interventional Science (Minimally Invasive Aesthetics), University College London (UCL), London, WC1E 6BT UK; 2 Crouch End Orthodontics, 72 Crouch End Hill, London, N8 8AG England UK; 3 Department of Plastic Surgery, Royal Free Hospital, University College London, London, UK

**Keywords:** Hyaluronidase, Hyaluronic acid, HA filler, Aesthetic medicine

## Abstract

**Background:**

Hyaluronidase is used as an adjunct or main treatment to manage complications associated with cosmetic hyaluronic acid (HA) filler injections such as necrosis, blindness, hypersensitivity, delayed nodules, and poor aesthetic outcomes.

**Objective:**

To systematically map the available evidence and identify the gaps in knowledge on the effectiveness of hyaluronidase use in managing the aesthetic complications associated with HA injections (vascular occlusion, blindness, nodules, delayed hypersensivity, granuloma, poor aesthetic outcome).

**Methods:**

PubMed, Medline, Embase and Cochrane databases were used up to May 2022, to look for randomized clinical trials (RCTs), clinical trials, and retrospective case-control studies reporting on the use of hyaluronidase for managing the HA filler injection complications.

**Results:**

The database search yielded 395 studies; of those 5 RCTs (all carried out in the USA) were selected (53 subjects), indicating the effectiveness of hyaluronidase for removal of un-complicated injected HA nodules (forearm, upper arm, or back skin). The follow-ups ranged from 14 days to 4 years. The amount of HA filler injected into each site varied from 0.2 to 0.4 mL. A dose dependent response was observed for most HA fillers. No major adverse reactions were reported. Overall, for removal of every 0.1 mL of HA filler they injected 1.25–37.5 units of hyaluronidase (single injections). When 3 consecutive weekly hyaluronidase injection was used much lower doses of 0.375–2.25 unit was utilised. There was no evidence in a form of RCTs, clinical trials, and retrospective case-control studies on the removal/reversal of HA injections in the facial skin, or management of over-corrections, inflammatory nodules, or tissue ischemia/necrosis associated with HA filler injection.

**Conclusion:**

Based on studies on the forearm, upper arm and back skin, hyaluronidase can be used for the reversal of uncomplicated HA filler injection nodule. However, further adequately powered studies are warranted to establish the ideal treatment protocol/dose of hyaluronidase for reversal of HA filler injections in the facial region or management of complications associated with aesthetic HA injection.

**Level of Evidence III:**

This journal requires that authors assign a level of evidence to each article. For a full description of these Evidence-Based Medicine ratings, please refer to the Table of Contents or the online Instructions to Authors www.springer.com/00266.

## Introduction

Cross-linked hyaluronic acid (hyaluronan, HA) is a polysaccharide; the most used reversible filler in aesthetic medicine [1, 2]. HA is a highly hydrophilic molecule and an essential part of the extracellular matrix; it attracts water molecules and creates a gel-like substance even at low HA concentrations contributing to the viscoelastic properties of the skin [1, 2]. HA is a polysaccharide consisting of the repeating disaccharide D-glucuronic acid and N-Acetyl-D-glucosamine units. Each disaccharide unit can bind up to 15 molecules of water, allowing 1 g of hyaluronan to bind about 16 l of water [2]. A high HA concentration, larger HA particle size, and increased number of cross-links all contribute to the increased durability of injectable HA fillers [1, 2]. Literature suggests that a high-molecular-weight HA has an anti-inflammatory behaviour, but a low-molecular-weight HA is pro-inflammatory [1–4].

Although HA injection is relatively safe [3, 4] for aesthetic procedures, complications can occur after HA injections, such as over-correction, or the Tyndall-effect (bluish discolouration caused by superficial injection of HA) [2], chronic oedema, inflammatory reaction, granuloma, vascular occlusion/compression, and tissue necrosis, to name a few. Early onset complications (<14 days post-injection) such as bruising, swelling, lumps, vascular occlusion, or tissue necrosis are mainly related to superficial placement, rapid injection/flow rates, or higher injection volumes [4–7].

Early signs of HA induced vascular occlusion can be immediate with severe pain, or temporary blanching that often lasts for a few seconds due to disruption of blood flow primarily beyond the injection site. Subsequently, pain can increase followed by bluish-red skin discolourations, and finally if left untreated, progression to tissue necrosis is inevitable [8, 9].

### Hyaluronidases

Hyaluronidase dissolves HA and contributes to HA metabolism; it is an enzyme that is found in bacteria, such as in *Staphylococcus aureus*, in venoms of bees and snakes, and in some spices’ testicular extracts [2]; Hyaluronidase cleaves HA’s β1,4-glycosidic bonds. Woodward et. al. [9] suggested that 30 Units of hyaluronidase were needed to dissolve 0.1 mL of HA.

The management of HA injection vascular complications is often with infiltration of the entire area with large volume of hyaluronidase (1500 units, every 2–8 hours) [8]. Hyaluronidase is administered directly at the HA injection site and along the course of the obstructed artery [10–12]. Hyaluronidase injection is more likely to be successful when performed early (< 4 h after injection of HA filler) [11]. Delay in hyaluronidase administration can lead to tissue necrosis, scarring, blindness, or in rare cases cerebrovascular accidents [13, 14]. Visual loss occasionally occurred in patients who received HA injections in the glabella, nose, or forehead and often presenting with sudden ocular pain, ptosis, and ophthalmoplegia [15]. Intra-arterial injection of 1500 units of hyaluronidase into the facial artery or supratrochlear artery successfully treated tissue necrosis in cases of vascular embolism after facial HA filler injection [16].

The incidence of delayed-onset nodules and inflammatory events is about 0.02c4.0% [4, 17–21]; they can appear months to years after the injection [4–7] and often require removal of the product [4–7]. Delayed inflammatory events are often type IV hypersensitivity reactions and T-cell mediated [4, 17–22]. A review of the delayed HA complications reported in the Manufacturer and User Facility Device Experience (MAUDE) FDA database [4] revealed that 33.3% were delayed onset, and of those, 71.8% were nodules (42.1% inflammatory and 29.7% non-inflammatory), 21.5% caused by hypersensitivity, and 6.7% were granulomas. Non-inflammatory nodules are treated mainly with hyaluronidase [22]. For inflammatory nodules, a combination of hyaluronidase, antihistamines, corticosteroids, anti-inflammatories, and antibiotics are often used. In case of hypersensitivity, a combination of corticosteroids, antihistamines, and anti-inflammatories are used. Granulomas are also treated with antibiotics and corticosteroids [22]. Overall, there is no universal protocol for the management of delayed onset nodules and inflammatory nodules.

### Mechanism of Action and Therapeutic uses of Hyaluronidase

Hyaluronidases are a group of 6 mucolytic enzymes that break down the HA [23] (table 1). They can be derived from mammalian tissue (animal-derived) that is often associated with impurities and their potential for allergic reactions (24, 25, 26), or synthesized in-vitro in pure form using recombinant technology (rHuPH20) [24].

**Table 1:** Different forms of hyaluronidase currently available in the market

Bovine sources	Hyaluronidase extracted from bovine testicular tissue	Amphadase^TM^ and Hydase^TM^
Ovine sources	Hyaluronidase extracted from ovine testicular tissue	Vitrase^TM^ or Hyalase®, a 1500. unit ampoule of powder for reconstitution (Wockhardt UK Ltd)
Human recombinant hyaluronidase	Non. animal based, pure form	Hylenex^TM^

For this review we briefly summarise the clinical uses of hyaluronidase as follows.

*A-Adjuvants to local anaesthesia to increase the duration and efficacy of anaesthesia in ophthalmic surgery *[25]*, and other fields, as well as* for chronic pain management and treatment of oedema or hematoma [23, 25].

Hyaluronidase has been used for retrobulbar, peribulbar, and sub-Tenon's anaesthesia in eye surgery, as a ‘spreading agent’. It reduces tissue oedema, *periorbital oedema,* and helps re-absorption of subcutaneous hematomas [2, 27, 28]. It assists vitrectomy, that is the surgery of the retina, where vitreous requires removing (the gel-like substance that fills the middle portion of the eye) and replacing it with another solution [2] by degrading hyaluronic acid into disaccharide compounds and dissociating intercellular substance barrier to liquefy the vitreous [2].

Hyaluronidase has been used to increase the duration and efficacy of anaesthesia; it hydrolysis the HA, increasing connective tissue permeability and collectively leading to a faster anaesthetic effect and a greater anaesthetized surface [28]. It has been used as an adjunct for inferior alveolar nerve blocks [29], intra-pulpal anaesthesia [30], or for craniectomy [31]. Hyaluronidase may also reduce pain associated with anaesthetic injections by decreasing tissue tension [28].

*B-Softening skin and related tissues, this includes topical treatment for phimosis (tight foreskin) *[32]*, prevention of perineal trauma (laceration) during delivery *[33]*, cervical ripening and induction of labour *[34]*, treatment of oral submucous fibrosis *[35]*,* a premalignant condition caused by betel chewing and can lead to squamous cell carcinoma, and *treatment of scleroderma-induced microstomia (limited mouth opening) *[36]*.* The sclerotic tissue found in systemic sclerosis is the result of the over-production of hyaluronan and collagen (types I, III, and VII) [36].

*C-Acceleration of insulin exposure,* hyaluronidase can be used in combination with rapid-acting insulin to accelerate insulin exposure, producing an ultra-rapid time-action profile with a faster onset and shorter duration of insulin action [37].

*D-Treatment of carpal tunnel syndrome* [38]

*E-Treatment of keloids and Hypertrophic scars* [39, 40]

*F-Off label use,* Hyaluronidase is commonly used for managing over-corrections, asymmetry, lumps, and nodules developed after HA filler injection. It dissolves subcutaneous nodules and degrades HA correcting excessive quantities of injected HA fillers [41–45] and its duration of the activity is about 24–48 h in dermal tissues [10]. It can be used at a high dose of 200 U or higher for treating skin necrosis associated with HA filler injections, caused by vascular occlusion and sometimes intra-arterially as a thrombolytic to treat eye/intracranial complications [16], and often without hypersensitivity test [23].

### Safety and Hypersensitivity

Intra-dermal injection of 3 units of hyaluronidase to test for the development of a wheal has been suggested [46, 47]. Hyaluronidase is contraindicated in patients who developed hypersensitivity reactions to bee or wasp stings, owing to the presence of hyaluronidase in the venom [46, 48, 49]. Relative contraindications are, the concurrent use of aspirin, corticosteroids, estrogens, furosemide, benzodiazepines, and phenytoin and anti-histamines. Above mentioned drugs may make tissues less sensitive to hyaluronidase and patients need a larger dose or repeated treatment [46, 47, 49].

The incidence of IgE-mediated Type I hypersensitivity is rare and estimated to be about 0.1%, unless large intravenous doses (> 200000 units) are given, giving a much higher incidence of 33% [13, 23]. In case of severe allergy caused by exogenous hyaluronidase, the use of autologous serum may be considered in non-acute cases requiring accelerated removal of HA filler [2]. Although rare, delayed allergic hypersensitivity reaction has also been reported after the treatment of granulomatous hyaluronic acid reaction [50].

### Study Objectives

The objective of the present scoping review was to systematically map the available evidence and identify the gaps in our knowledge on the effectiveness of hyaluronidase use in managing the aesthetic complications associated with HA injections (vascular occlusion, blindness, nodules, delayed hypersensivity, granuloma, poor aesthetic outcome) and to report on the adverse effects associated with the use of hyaluronidase.

## Methodology

For this scoping review, we used the PRIRMA extension for scoping reviews [51] and provided an overview or map of the evidence that was available. Search Strategy included the PubMed, Medline, Embase, and Cochrane Library databases. They were searched up to May 2022 for randomized clinical trials (RCTs), clinical trials, and retrospective case-control studies that assessed the efficacy of hyaluronidase in managing the complications of aesthetic HA injections. The search and MeSH terms are summarised in tables 2 and 3. The reference lists of all included studies and previous reviews were also searched for additional RCTs, clinical trials, and retrospective studies. We looked for the parameters such as patient’s age, gender, indication for hyaluronidase injection, injection site, dose (hyaluronidase units), the brand names used for hyaluronidase and HA filler, follow-up after hyaluronidase injection, main study findings, as well as reported adverse effects. The PICOS approach was used as follows,

**Table 2:** Keyword search and MeSH terms used for PubMed search for the present scoping review.

Concept 1	"adverse effect*"[tw] OR nodule*[tw] OR papule*[tw] OR mass*[tw] OR lump*[tw] OR bump*[tw] OR induration*[tw] OR granuloma*[tw] OR "granulation tissue*"[tw] OR "foreign bod*"[tw] OR hypersensitivity*[tw] OR swelling*[tw] OR inflammation*[tw] OR thrombosis[tw] OR ischemia[tw] OR embolism**[tw] OR "vascular embolism*"[tw] OR "vascular occlusion*"[tw] OR necrosis[tw] OR blindness[tw] OR dermatosis[tw] OR "adverse event*"[tw] OR complication*[tw]
	*MeSH: "Visual Acuity"[Mesh] OR "Blindness"[Mesh] OR "Embolism"[Mesh] OR "Intracranial Embolism"[Mesh] OR "Embolism and Thrombosis"[Mesh] OR "Intracranial Embolism and Thrombosis"[Mesh] OR "Ischemic Stroke"[Mesh] OR "Edema"[Mesh] OR "Ecchymosis"[Mesh] OR "Skin Diseases, Vascular"[Mesh] OR "Skin Diseases, Papulosquamous"[Mesh] OR "Skin Diseases, Eczematous"[Mesh] OR "Skin and Connective Tissue Diseases"[Mesh] OR "Skin Diseases, Bacterial"[Mesh] OR "Skin Diseases, Viral"[Mesh] OR "Pain"[Mesh] OR "Chronic Pain"[Mesh] OR "Eye Pain"[Mesh] OR "Hypersensitivity"[Mesh] OR "Hypersensitivity, Delayed"[Mesh] OR "Drug Hypersensitivity"[Mesh] OR "Vasculitis, Leukocytoclastic, Cutaneous"[Mesh] OR "Inflammation"[Mesh] OR "Vascular Diseases"[Mesh] OR "Injections, Intra. Arterial"[Mesh] OR "Postoperative Complications"[Mesh]*
Concept 2	"Hyaluronic Acid"[tw] OR Hyaluronoglucosaminidase[tw] OR "HA filler*"[tw] OR "Cosmetic Techniq*"[tw] OR "Dermal Filler*"[tw]
	*MeSH: "Hyaluronic Acid"[Mesh] OR "Cosmetic Techniques"[Mesh] OR "Dermal Fillers"[Mesh] OR "Injections, Subcutaneous"[Mesh]*
Concept 3	*Hyaluronidase[tw] OR *HYAL[tw] OR* Hylenex[tw] OR Hydase[tw] OR Vitrase[tw] OR Wydase[tw] OR Hyalase[tw]*
	*MeSH: "Thrombolytic Therapy"[Mesh] OR "Hyaluronoglucosaminidase"[Mesh]*
Concept 4	"RCT*"[tw] OR "Randomi#ed clinical trial*"[tw] OR "clinical trial*"[tw] OR "retrospective stud*"[tw] OR “Case. Control Stud*"[tw] OR “Case Control Stud*"[tw]
	*MeSH: "Retrospective Studies"[Mesh] OR "Prospective Studies"[Mesh] OR "Follow. Up Studies"[Mesh] OR "Case. Control Studies"[Mesh]*

**Table 3:** Keyword search and MeSH terms used for Ovid medline for the present scoping review

1.	Hyaluronic acid/
2.	Cosmetic techniques/
3.	Dermal fillers/
4.	Injections, subcutaneous/
5.	(Hyaluronic Acid or Hyaluronoglucosaminidase or HA filler* or cosmetic techniq* or dermal filler*).mp. [mp=title, abstract, heading word, drug trade name, original title, device manufacturer, drug manufacturer, device trade name, keyword heading word, floating subheading word, candidate term word]
6.	1 or 2 or 3 or 4 or 5
7.	Postoperative complications/
8.	Injections, intra. arterial/
9.	Vascular diseases/
10.	Inflammation/
11.	Vasculitis, Leukocytoclastic, Cutaneous/
12.	Drug hypersensitivity/
13.	Hypersensitivity, delayed/
14.	Hypersensitivity/
15.	Eye pain/
16.	Chronic pain/
17.	Pain/
18.	Skin diseases, viral/
19.	Skin diseases, bacterial/
20.	Skin.mp. and connective tissue diseases/[mp=title, abstract, heading word, drug trade name, original title, device manufacturer, drug manufacturer, device trade name, keyword heading word, floating subheading word, candidate term word]
21.	Skin diseases, eczematous/
22.	Skin diseases, Papulosquamous/
23.	Skin diseases, vascular/
24.	Ecchymosis/
25.	Ischemic stroke/
26.	Edema/
27.	Intracranial Embolism.mp. and Thrombosis/[mp=title, abstract, heading word, drug trade name, original title, device manufacturer, drug manufacturer, device trade name, keyword heading word, floating subheading word, candidate term word]
28.	Embolism.mp. and Thrombosis/[mp=title, abstract, heading word, drug trade name, original title, device manufacturer, drug manufacturer, device trade name, keyword heading word, floating subheading word, candidate term word]
29.	Intracranial embolism/
30.	Embolism/
31.	Blindness/
32.	Visual acuity/
33.	(adverse effect* or nodule* or papule* or mass* or lump* or bump* or induration* or granuloma* or granulation tissue* or foreign bod* or hypersensitivit* or swelling* or inflammation* or thrombosis or ischemia or embolism* or vascular embolism* or vascular occlusion* or necrosis or blindness or dermatosis or adverse event* or complication*).mp. [mp=title, abstract, heading word, drug trade name, original title, device manufacturer, drug manufacturer, device trade name, keyword heading word, floating subheading word, candidate term word]
34.	7 or 8 or 9 or 10 or 11 or 12 or 13 or 14 or 15 or 16 or 17 or 18 or 19 or 20 or 21 or 22 or 23 or 24 or 25 or 26 or 27 or 28 or 29 or 30 or 31 or 32 or 33
35.	Hyaluronoglucosaminidase/
36.	Thrombolytic therapy/
37.	(Hyaluronidase or HYAL or Hylenex or Hydase or Vitrase or Wydase or Hyalase).mp. [mp=title, abstract, heading word, drug trade name, original title, device manufacturer, drug manufacturer, device trade name, keyword heading word, floating subheading word, candidate term word]
38.	35 or 36 or 37
39.	Retrospective studies/
40.	Case. Control studies/
41.	Follow. Up studies/
42.	Prospective studies/
43.	(RCT or Randomi#ed clinical trial* or clinical trial* or retrospective stud* or Case. Control stud* or Case control Stud*).mp. [mp=title, abstract, heading word, drug trade name, original title, device manufacturer, drug manufacturer, device trade name, keyword heading word, floating subheading word, candidate term word]
44.	39 or 40 or 41 or 42 or 43
45.	6 and 34 and 38 and 44

Participants,

- Patients who received hyaluronic acid (HA) for aesthetic interventions and developed complications (necrosis, blindness, hypersensitivity, delayed nodules, poor aesthetic outcome) and treated with hyaluronidase 
- Non-complicated subjects who received HA followed by hyaluronidase to assess the effectiveness of hyaluronidase in removing/degrading HA

***Intervention***, hyaluronidase

***Comparison***, placebo, or control (received HA filler, but no hyaluronidase)

***Outcome***, improvement in aesthetic complications, or changes in HA site characteristics (diameter of the HA filler nodule, elevation, firmness), we also provide a report on adverse effects associated with hyaluronidase use (hypersensitivity reactions, facial angioedema, and anaphylaxis)

***Study type***, randomised clinical trials (RCT), clinical trials, retrospective case-control studies

## Results

### Search Results

The database search yielded 395 studies (PubMed, Medline, Embase, and Cochrane Library databases). After reviewing the titles and abstracts, authors identified 5 RCTs 4 papers [52–55] (Table 4) that were relevant to this scoping review. Overall, 5 RCTs with 53 subjects reported the effectiveness of hyaluronidase for eliminating uncomplicated HA nodules. All studies were carried out in the USA.

**Table 4:** The summary of the findings of the included studies in the present scoping review

Authors, country, and funding	Follow up	Study sample study type	Type of hyaluronidase & dose	Type of hyaluronic acid & dose	Inclusion criteria complication	Outcome adverse effects
Vartanian et. al. [52] USA Funding: nothing mentioned	4 months	Randomized controlled trial *n*=12 Randomly received hyaluronidase or normal saline (control) 7 female, 5 male Mean age=43.7 years (30. 60 yrs. old) Palpation scores* (0. 4) were recorded at days 1. 3, 4. 7, 8. 14, 15. 28, 29. 60, 61. 90, and 91. 120 after injection with hyaluronidase or saline	No brand name mentioned Each site received 0.5 mL hyaluronidase injection (75 units in normal saline), 1–3 days after HA gel injection 30. gauge needle was used	Restylane (Q. Med AB, Uppsala, Sweden) 0.2. mL of HA gel injected at each site 30. gauge needle was used Injected into the upper to middle dermis	Uncomplicated HA nodule Forearm (ipsilateral, 2 sites, 5 cm and 15 cm proximal to the wrist) The grading physician and patients were blinded to the post. injection substance	After a week, 80% reduction observed in nodule size in the hyaluronidase group vs. 10% in saline controls Suggested an initial injection dose of 5. 10 U of to avoid allergic reaction 2 weeks after HA injection 4 patients who received hyaluronidase injection developed localized hypersensitivity reactions (site redness and mild pruritus)
Vartanian et. al. [52] USA Funding: nothing mentioned		Randomized controlled trial *n=*8 5 female, 3 male Mean age=38.1 years Palpation scores (0. 4) recorded	10, 20, or 30 units 0.4 mL injection	Restylane (Q. Med AB, Uppsala, Sweden) 0.2. mL of HA gel injected at each site	Uncomplicated HA nodule Forearm (3 sites, 5, 10, and 15 cm to the wrist) Injection site doses were selected randomly	No statistically significant difference between doses Mild localized allergic responses in 2 (25%) subjects
Juhász et. al. [53] USA Funding: nothing mentioned	14 days	Blinded randomized study *n=*15 mean age=27.2 years Female = 53.3% Male = 46.7% Blinded palpation done on day 0, 1, 4, 7, 14 5. point ordinal grading scale (0. 4) was used (Vartanian et. al., 2005)	Valeant (Laval, Canada) On the same day of HA injection, each site received either no injection, normal saline (0.2 mL), 20 units, or 40 units of hyaluronidase. Procedure repeated once. 30 gage needle was used	7 brands tested JUV_X_, JUV_X_ & JUV_V_ (Juvederm, Allergan, Irvine, CA) RES_L_, RES_S_ & RES_LYFT_ (Restylane, Galderma Laboratories, Lausanne, Switzerland) BEL (Merz Aesthetics, Greensboro, NC) 0.2 mL bolus technique 27. gauge needle was used Injected into the upper to middle dermis	Uncomplicated HA nodule Experiment carried out on back skin with patients lie in a prone position 28 sites (3 cm apart)/patient Sites randomly received 0.2 mL of each brand of HA (each brand used in 4 sites) blinded palpation score recorded using an ordinate, qualitative palpation scale.	Palpation scale decreased (*p* < 0.05) at days 1, 2, 3, 4, 7, and 14 post. injections for both 20 and 40 units of hyaluronidase, compared with sites that were not injected or injected with normal saline only 20 and 40 units of hyaluronidase were equally effective in dissolution of all HA fillers BEL was the fastest HA filler to degrade. RESS (high cross. linking bonds and HA concentration) was the slowest to dissolve over the 14. day period 20 units of hyaluronidase for every 4 to 6 mg of HA was suggested. There were tenderness over the injection sites (*n* = 6), itching of the back skin after injection (*n* = 2), and minimal bruising (*n* = 1), but all were self. resolving within 2. 3 days.
Alam et. al. [54] USA Funding: Departmental research funds, no company mentioned	4 months Patients followed 1 week after the third & final hyaluronidase injection and then for final follow. up 3 months later Based on “visual detection” & “palpability score” using a 5. point scale (0. 4) Patient and clinician recorded scores	randomized clinical trial Split. arm, parallel. group RCT *n*=9 All women Mean age=45.8 (15.7) years	Vitrase (Alliance Medical Products, Inc) At 1, 2 & 3 weeks after HA injection, each site injected with a 0.1 mL of variable. dose hyaluronidase (1.5 U, 3 U, or 9.0 U) or saline (control) Experiment Repeated 1, 2, and 3 weeks after HA filler placement. Small volume (0.1 mL) injection of very low doses (1.5. 9 unit) of saline. diluted hyaluronidase 30. gauge needle Was used	Restylane. L (Galderma Laboratories) & Juvéderm Ultra XC, (Allergan Inc) 0.4 mL each site	Uncomplicated HA nodule Bilateral upper inner arms Each subject had 8 injection sites along their bilateral upper inner (medial) arms, 4 on each arm, 8 sites/patient	injection of small. volume (0.1 mL) of very low doses (9 unit) of hyaluronidase at weekly intervals was effective Higher dose of hyaluronidase was needed to reverse Juvéderm Ultra XC compared to Restylane. L All filler sites decreased in terms of visual detection & palpation over time. Normal saline (control) & hyaluronidase were different at 4 weeks & 4 months (*p*<0.05) This was also mirrored in subjects’ self. assessment at 4 weeks & 4 months (*p*<0.05) The 9 units hyaluronidase injections were less palpable than the 1.5 units injections at both 4 weeks & 4 months Differences were more notable for Restylane. L than for Juvéderm Ultra XC No adverse events reported.
Zhang. Nunes et. al. [55] USA Funding: Unrestricted grant to the USC Department of Ophthal. mology from Research to Prevent Blindness, New York, NY No company mentioned	4 years Diameter of the HA filler nodule (mm), elevation & firmness (both scored using a Likert. type scale 0,1,2,3) were measured pre. injection & at varying time points Time points: Immediately after injection of HA gel, one week prior to hyaluronidase injection, immediately after hyaluronidase injection & at 15 min, 30 min, 1 h, 2 h, 3 h, 5 h, 8 h, 1 day, 2 days, 3 days, 1 week, 2 weeks, 1–2 months, 5–6 months, 1 year, 2 years & 4 years	Randomized clinical trial (*n*=9) All women Mean age = 45.8 (15.7) years Subjects, graders & injectors were masked.	Hylenex (recombinant human hyaluronidase) One week after HA injection, each site received its randomized equal volumes (0.15 mL) of 2.5 U, 5 U, 10 U, 20 U of hyaluronidase, saline, or nothing, the seventh site was used as a control for 10 U of hyaluronidase only (to assess the adverse effects, hypersensivity.	Restylane. L Juvéderm Ultra Juvéderm Voluma 0.2 mL each Site	Uncomplicated HA nodule Forearm 7 sites per forearm 18 forearms 6 sites received HA One site only hyaluronidase (control)	The most notable changes for all fillers occurred between the 30. min and 3 h time points, and followed by continued gradual degradation through week 2. There were no allergic reactions; 3 subjects had significant erythema & minimal irritation of the HA sites starting five hours after initial intradermal implantation & lasting up to 1–2 days. This irritation was most significant with Restylane. Some mild post. inflammatory hyperpigmentation was observed, which resolved after 3 months.

*A score of 0–4 given as follows; 4 for maximal augmentation; 3 for moderately raised; 2 to a slightly raised bump; 1 to sites that were barely palpable at examination; and 0 to sites with no tactile trace of injected HA gel [52].

### Effectiveness of Hyaluronidase for Removal of Uncomplicated HA Nodules

The follow-ups for 5 clinical trials ranged from 14 days to 4 years. The amount of HA filler injected into each site varied from 0.2 to 0.4 mL per site. The injected hyaluronidase doses were 1.5–75 units per site. Overall, for removal of every 0.1 mL of HA filler the reviewed studies suggested an injection of 1.25–37.5 units of hyaluronidase (single injections). When 3 consecutive weekly hyaluronidase injection were used much lower doses of 0.375–2.25 unit was utilised.

The HA brands tested were Restylane (Q-Med AB, Uppsala, Sweden), JUV_X_, JUV_X_ & JUV_V_ (Juvederm, Allergan, Irvine, CA), RES_L_, RES_S_ & RES_LYFT_ (Restylane, Galderma Laboratories, Lausanne, Switzerland), BEL (Merz Aesthetics, Greensboro, NC), Juvéderm Ultra XC, (Allergan Inc), Restylane-L (Galderma Laboratori), and Restylane-L, Juvéderm Ultra & Juvéderm Voluma. They tested the products on ipsilateral forearms, bilateral upper inner (medial) arms, and back skin. The brand of hyaluronidase used for these studies was non-specified [52] in one study; Valeant (Laval, Canada), Vitrase (Alliance Medical Products, Inc) and Hylenex (recombinant human hyaluronidase) were used in other studies [53–55]. Mean age and gender were reported for all 5 selected studies.

Overall, 5 RCTs with 53 subjects, confirmed the effectiveness of hyaluronidase for removal/reversal of un-complicated injected HA nodules in the forearm, upper arm and back skin and no major adverse reactions were reported. Vartanian et. al. [52] did not observe a significant difference between various doses of hyaluronidase (10, 20, or 30 units) at 4 months; this study didn’t have a control group (without hyaluronidase injection) and some trends were also seen for the effectiveness of higher doses (30 units) at some time points (1–3 days, 8–14 days, and 29–60 days) [52]. Juhász et. al. [53] reported no significant difference in efficacy and rate of dissolution of all HA filler between 20 and 40 units of hyaluronidase. However, some trends were identified such as, the Restylane Silk was the slowest to dissolve over the 14-day period, contrary to the Belotero Balance, which was the fastest to degrade.

Alam et. al. [54] used 3 consecutive doses of hyaluronidase (1.5, 3, 9 Units) at 1, 2, and 3 weeks after HA filler injection, and observed that repeated weekly low-volume (1mL), low-doses of hyaluronidase (9 units) is effective, in particular for correction of minor irregularities. They noted a dose dependent response, with 9.0-unit hyaluronidase injection being more effective than 1.5-unit sites at 4 weeks and 4 months.

Zhang-Nunes et. al. [55] studied sites injected with Restylane- Lyft, Juvéderm Ultra, and Juvéderm Voluma for 4 years after hyaluronidase injection (2.5, 5, 10, or 20 units). Most of the changes for all HA filler injected sites occurred between the 30 min and 3 hr time points, after hyaluronidase injection, that continued gradual degradation up to day 3, with minimal change through week two. All 3 tested fillers showed a dose response, with Voluma being more pronounced. Juvéderm Voluma required higher doses of hyaluronidase for dissolution (20 units), and Restylane appeared to respond to lower doses of hyaluronidase (2.5 units).

The present scoping review did not identify similar evidence for HA filler reversal in the facial region.

### Effectiveness of Hyaluronidase for Management of Over-Corrections, Inflammatory Nodules, or Vascular Occlusions Following Aesthetic HA Filler Injections

Similarly, no evidence was found, in a form of RCTs, clinical trials, and retrospective case-control studies, on the management of over-corrections, inflammatory nodules, or tissue ischemia/necrosis associated with aesthetic HA filler injections; the available evidence was mainly retrospective case reports or case series. We have also listed the summary of 11 excluded studies [10, 14, 16, 56–63] in table 5. They were retrospective case series, with 5 cases or more, reporting on the use of hyaluronidase for managing aesthetic complications associated with aesthetic HA filler injection. This includes management of delayed onset nodules in tear trough augmentation [56], lip augmentation [59], delayed peri-orbital oedema [61, 62], aesthetic over-correction [10], Tyndall effect, and late nodules [10], late inflammatory cutaneous reactions in lip and tear trough regions [58], as well as management of skin necrosis [14, 57], and vascular complications with [60, 63] or without visual impairment [58].

**Table 5:** List of the selected excluded studies and their summary findings

Authors	Follow. up	Study sample Study type	Hyaluronidase ± other medicines	Type of hyaluronic acid dose	Inclusion criteria complication	Outcome adverse effects
Hilton et. al. [56]	Not clear	Retrospective study (*N*=20) Case series No control 3 male, 17 female. Age range= 32–74 years old mean age= 49.3 years old	20. 75 units (Hylase Dessau®). Diluted in 1.0 mL of normal saline. 0.2–0.5 mL injection	HA brand not reported	Nodule developed after HA tear trough augmentation. Infraorbital Region. 14 patients had HA injection for tear. trough augmentation. 6 patients had no HA injection and idiopathic edema	79% of patients had full nodule resolution with one injection. 3 patients (21%) required an additional injection. No adverse effect observed. There was a loss of original HA treatment effect in two cases (14.3%)
Sadeghpour et. al. [59]	Not clear	Retrospective study (*n*=5)No control Case seriesOriginal sample of 1,029 patient All femaleAge range 46. 70 years old	All patients treated first with hyaluronidase (Hylenex, no dose given) without significant clinical improvement. Then, a combination of triamcinolone (2. 5 mg/mL) & hyaluronidase (multiple treatment sessions) given.	1,250 Vycross HA filler treatments (1,029 patients) Juvederm Volbella Juvederm Vollure Juvederm Voluma	Delayed. onset nodules (lip) developed in 5 cases (Juvederm Volbella) after 35.8 week on averageSubcutaneous, firm, non. erythematous, and nontender nodule	Combination of triamcinolone and hyaluronidase (multiple treatment sessions) lead to diminution in nodule size.All patients were treated successfully with near. complete or complete resolutionNo mention of adverse effects
Bravo et. al. [10]	Not clear	Retrospective study (*n=*114)Case seriesNo control 112 females, 2 malesMean age = 43 years old	HYAL (hyaluronidase 2.000 IU, BIOMETILtm, dissolved in 5 ml of the diluent, saline solution, water for injection & mannitol) 400 IU/ml solution 2. 2000 UI injected with a mean dose of 68 UI	HA brand not reportedHA injections were as follows: Eyelid region (*n=*58) Upper lip (*n*=10)Lower lip (*n=*9)Malar region (*n*=9) Melomental fold (*n*=5) Forehead (*n*=4)Nose (*n*=4)Chin (*n*=4) Pyriform aperture (*n*=3) Temple (*n*=2)Lateral orbit (*n*=2) Zygoma (*n*=2)Peri. labial (*n*=1) Acne scar (*n*=1)	Over. correction (45%)Tyndall effect (44%)Late nodules (11%)	40 UI of hyaluronidase injection should be enough for each CM2 of HA that needs removalThe main complications were moderate edema and local burning sensation.One case developed lip angioedema and treated with 80 mg oral prednisolone. No dermal or subcutaneous atrophies were observed
Skippen et. al. [61]	Mean follow. up = 11 months (6. 20 months)	Retrospective study *n*=61Case seriesNo control 59 female, 2 male age range = 21. 78 years mean age=48 years old	HYAL15. 90 IU per eyelid	Had different types of HAHA brand not reported	Had lower eyelids or cheeks HA injection and developed Lower eyelid edema On average 3 years (1. 10 years) after HA filler injection	Single hyaluronidase injection was effective in 92% of patients 8% required another hyaluronidase injection to completely eradicate residual edema6 patients (10%) were satisfied after hyaluronidase only 6 patients (10%) had lower eyelid blepharoplasty. 48 patients (80%) had secondary treatment with HA filler injection No adverse effects mentioned
Dubinsky. Pertzov et. al. [62]	The mean follow. Up = 10.85 months (6. 36 months)	Retrospective study *n*=17Case seriesNo control Female Age range = 26. 80 years oldMean age=54.9 years old	HYAL3. 90 IU per eyelid	Had different types of HAHA brand not reported	Upper eyelid edema (periocular edema) 6. 24 months after HA filler injection in the supraorbital area.	Edema resolved in all patients after hyaluronidase treatment13 (76.4%)were satisfied and didn’t receive HA filler again4 received additional HA filler.No mention of adverse effects
Artzi et. al. [58]	Not clear	Retrospective study *n=*17No controlCase seriesOriginal sample of 400 patients and 17 developed complications	17 patients were first treated with oral ciprofloxacin (500–750 mg, bd, for 3. 4 weeks), which treated 6 patients with no recurrence11 patients had recurrent episodes and received broad. spectrum antibiotics (ciprofloxacin or rifampicin for a minimum of 3 weeks) and multiple intralesional injections of hyaluronidase (30–100 unit/mass or nodule)	400 patients (360 females, 40 males; average age = 49.6 years) received Juvéderm Volbella (HA. Vb) filler into the tear trough area (*n*=351) or lips (*n*=49).Other HA. fillers were also used in other areas of the face	Late inflammatory cutaneous reactions in tear trough area & lips17 (4.25%) patients 13 in tear trough area (n. 13) and lips (*n*=4)Patients developed prolonged (up to 11 months) and recurrent (average: 3.17 episodes) late inflammatory cutaneous reactions (average onset: 8.41 weeks following HA filler injection, range: 5–12 weeks)	All symptoms & signs receded in 6 patients who received initially antibiotics and did not recur.Recurrent episodes (11 patients) were treated with repeated courses of broad. spectrum antibiotics and multiple intralesional injections of hyaluronidase. Oral, intramuscular & intralesional administration of corticosteroids were less effective.No mention of adverse effects
Sun et. al. [57]	Not clear	Retrospective study *n*=20Case control study20 consecutive referrals with nasal skin necrosis following nose and/or nasolabial fold augmentation with HA fillersAge range 21. 52 years old19 female, 1 male	18 received hyaluronidase [ovine testicular hyaluronidase (Shanghai First Biochemical Pharmaceuticals Corp., Shanghai; equivalent to Vitrase in North America, 150 to 300 units] from 0 hrs. 7 days after developing skin problemsAlso given antibiotics, tanshinone, papaverine, topical magnesium sulphate, infrared irradiation, and hyperbaric oxygen. Patients were also given aspirin, unless contraindicated.	HA brand not reportedHA was injected into the nasion area (*n*=14), nasolabial fold (*n*=5) or both (*n*=1)	Skin necrosis after HA injection in nose, nasolabial fold, glabella & forehead, or lip	7 patients (%35) developed skin necrosis.13 patients (%65) recovered fully following combination treatment with hyaluronidase.Early (<2 days) combination treatment with hyaluronidase was associated with the full resolution of the complicationsNo mention of adverse effects
Ors [14]	Followed up for 3–24 months	Retrospective study *n*=16No controlCase seriesAge range= 18. 60 years oldAll female	6 patients only received palliative treatment 10 patients received 1500 units (300 U with every 4. 6 hrs) hyaluronidase [30 s after HA injection (*n*=4) or 6. 24 h after HA injection] and palliative treatment.Patients with open wounds received topical Fusidic acid antibiotic. Patients with large wounds received amoxicillin–clavulanate orally for 14 days.	HA brand not reportedoverall, 841 cases received HA filler Nasolabial area (391)Lip (225)Glabella–forehead (90)Infraorbital (46)Malar region (25)Chin (24) Nose (40)	Vascular occlusion. related ischemia and skin necrosis after HA injectionComplications occurred in 16 cases [Nose (*n*=4), Nasolabial area (*n*=4), Glabella (*n*=8) area]Skin complications presented in 4 patients during HA injection and in 12 patients 6–24 h after the procedure.	Recovery time for patients who received palliative treatment and had limited necrosis was 40. 60 days, without obvious scar.Patients who received hyaluronidase and palliative treatment, had larger necrosis areas, and at the end a large necrosis area was observed in 4 patients. The recovery period in this group was 20. 90 days.Overall, 4 patients healed with a scar and 12 healed without any significant scar.Authors suggested Hyaluronidase injection provides earlier recovery of limited skin necrosis. Immediate hyaluronidase injection allowed small damage to heal in a short time; however, it did not eliminate large necrosis, but limited the necrotic area’No adverse effect observed
Zhang et. al. [16]	Average follow. up = 5 months (3. 12 months)	Retrospective study *n*=17No controlCase series19. 52. year. oldMean age=33 years old14 female, 3 male	13 patients had 1500 IU hyaluronidase (Shanghai Shangyao No. 1 Biochemical Pharmaceutical Co., LTD.) injected into facial artery 4 patients had 1500 IU hyaluronidase injected into supratrochlear artery Received intra. arterial thrombolysis therapy 2 hours to 7 days after the onset of symptomsPatients also received;1. glucocorticoid pulse therapy (dexamethasone sodium phosphate, 10 mg, ivgtt, qd, 3 d)2. neurotrophic treatment (mecobalamin injection, 0.5 mg, ivgtt, qd, 90 d)3. anti. allergy treatment (loratadine, 10 mg, pol, qd).	HA brand not reportedHA filler was injected in the nasolabial fold (*n*=7) or forehead (*n*=5)	Vascular complications of HA fillerAll had skin necrosis or skin ecchymosis spreading along the surface branches of facial artery or supratrochlear artery4 had severe ptosis and weakness in eye opening, ocular pain, and skin numbness. 2 had ocular motility disordersNone had visual impairment and life. threatening injuries (hypertension, coagulopathy, intracranial and external haemorrhaging)The colour doppler flow imaging was used to locate the facial and supratrochlear arteries	The skin necrosis of 16 patients was completely healed and 1 patient left with small, superficial scarsThe ptosis and ocular motility were completely resolved.No mention of adverse effects
Zhang et. al. [60]	Average follow. up = 3 months (1 month to 1 year)	Retrospective study *n*=24no controlCase seriesMean age=26 years old23 female, 1 male	11 patients received intra. arterial injection of hyaluronidase (500. 1500 units) 13 patients received intra. arterial injection hyaluronidase (750. 1500 units) and urokinase (100,000. 250,000 units)	HA brand not reportedHA was injected into the nasion area (*n*=12), followed by the frontal (*n*=10), glabella (*n*=1) & temporal (*n*=1).	Severe visual impairment (unilateral, 10 right & 14 left eyes were affected) after cosmetic facial hyaluronic acid injectionsAll had ptosis & mydriasis with pupil diameter larger than 5 mm 22 patients had ocular motility disorders. 20 patients had skin lesions in the corresponding region of artery occlusion, being pale, piebald, or necrotic.None had life. threatening injuries or interventional contraindications (hypertension, coagulopathy, intracranial & external haemorrhaging)	10 (42%) patients had improved visual acuity after treatment, 14 patients had no visual impairment improvement The ptosis in 24 patients were all healed. 3 patients had secondary embolization, sudden headache, ocular pain, and decline in vision on the 1st or 2nd day after the first intraarterial thrombolysis therapy and had 2nd course of intraarterial thrombolysis therapy with less success. Partial or total ocular motility limitation healed in 8 patients and ameliorated in 14 patients Intra. arterial thrombolysis therapy significantly improved skin necrosis and ecchymosis, and nearly restored the patients’ appearance to normal, leaving only some superficial scars in several patients.Authors suggested that combined hyaluronidase and urokinase may have a better thrombolysis effect on hyaluronic acid embolismIn addition, early hormone shock (high. dose glucocorticoid), local hyaluronidase injection, oxygen inhalation, Mannitol, and melilotus extract tablets were necessary to reduce tissue edema.Earlier treatment was associated with the higher vision improvementNo mention of adverse effects
Wang et. al. [63]	30 days follow. up	Retrospective study *n*=30Case seriesNo control 1 male, 29 femalesAge range= 18. 35 years old	30 had local injection of hyaluronidase at the HA injection site immediately after the onset of vision loss and 25 received retroocular hyaluronidase injection at other hospitals.28 had one and 2 patients had 2 intra. arterial thrombolysis(Using microcatheter, 1500 units of hyaluronidase diluted in 1 mL of saline and were slowly infused followed by infusion of 1 mL papaverine diluted in 2 mL saline)	HA brand not reported	Presented 20. 120 hrs after the onset of blindness (median, 59.5 h).Had monocular blindness due to HA injection in the forehead (*n*=3) and nasal bridge (*n*=27) regions 26 had ptosis 23 had ocular motility disorders. 3 had light perception 27 had no light perception with varying degrees of vascular occlusion in the retina	The ptosis disappeared and 18 patients had normal eye movement after the intra. arterial therapy.5 patients had visual improvement 4 patients with complete vision loss gained some light perception.

The selected excluded studies used a wide range (2–2000 units) of hyaluronidase doses for treatment of delayed non-inflammatory HA nodules and over-corrections [10, 56, 59]. Two studies [10, 56] only used hyaluronidase and the other one [59] used triamcinolone (2–5 mg/mL) in combination to hyaluronidase. Of particular interest was the study by Sadeghpour and colleagues [59], indicating higher incidence of delayed non-inflammatory HA nodules in HA fillers used Vycross technology. Two studies [61, 62] reported on edema management of upper and lower eyelid and tear trough area that used 3–90 units of hyaluronidase per eyelid to remove the HA filler. Lower eyelid and tear rough area appears to be a challenging area for HA filler removal or corrections as reflected by the %80 need for HA filler reinjection and 10% need for blepharoplasty in this area [61]. For upper eyelid only about 25% of patients needed HA filler reinjection [62].

## Discussion

As explained earlier, based on the limited available evidence, a scoping review of the literature was conducted [51], suggesting a paucity of studies assessing the efficacy of hyaluronidase for the reversal of HA injections in the facial region. The findings were based on studies with small sample sizes and the experiments on the forearm, upper arm, and back skin [52–55]. The applicability of findings generated from the study of arms (volar forearm or upper arm) or back skin for products designed for the face can be questionable [64, 65]. This is due to the varying thickness and elastic properties of the skin in different anatomical regions [65], which can be influenced by variations in components such as elastic network, fibrous collagen, and glycosaminoglycans [64–66].

There were some variations in the degree of degradation of HA fillers after hyaluronidase injection that could be partly explained by the HA particle size, degree of cross-linking, different cross-linking technologies, and the amount of G-prime [2]. We know from research [2] that higher HA concentration (such as JUV family), increased amount of G-prime (JUV X+ and RES LYFT), larger HA particle size (such as RES LYFT), and increased number of cross-links (such as in JUV X and JUV X+ using Hylacross technology) contribute to increased durability of injectable HA fillers requiring higher dose of hyaluronidase for dissolution. However, this effect was mainly seen in the in the first 2–3 weeks following hyaluronidase injections.

For this scoping review, we did not include animal studies, and studies that included other dermal fillers such as silicone [67] or calcium hydroxyapatite [68]. Congress abstracts [69], case reports or case series reporting less than 5 cases [70] were excluded. A review of the selected excluded studies (> = 5 subjects) highlighted some interesting findings, suggesting that hyaluronic acid injections into the glabellar region, nasal dorsum, and nasolabial fold near the eyes were the main sites with serious vascular occlusions and eye problems that needed urgent treatment [14, 16, 57, 60]; some authors suggest the injection in these sites is not advised [71, 72]. Subsequent to vascular occlusion, patients often develop pain and characteristic reticulated erythema [57] in the areas supplied by facial artery [48, 73, 74]. The most common initial ocular symptoms after HA injection are vision loss (%50) and orbital pain (%35) as well as headache and dizziness (%26) [75].

Different methods of hyaluronidase injection (localised or intra-atrial) have been used to manage the vascular occlusions such as skin necrosis, eye movement and visual impairment disorders, ptosis, as well as blindness or loss of visual field. In order to treat skin necrosis, some researchers used localised infiltration of hyaluronidase at the HA injection site [14, 57] and some utilised intra-arterial injection of the either facial artery or supratrochlear artery [16]. It appears that immediate [14] or early treatment (< 2 days) [57] with localised hyaluronidase injection or intra-arterial injection [16] is associated with better outcome and full resolution of the skin necrosis. Zhang et. al. [16] described the ideal location for intra-arterial injection of hyaluronidase, as the junction of the lower margin of the mandible and the anterior margin of the masseter muscle. The facial artery diameter at this point is about 2.3 mm and less than the needle diameter (0.57–0.92 mm) [16]. Similarly, for management of visual impairments, local injection of hyaluronidase at the HA injection site [63], retroocular hyaluronidase injection [63], or intra-arterial injection [16, 60, 63] as well as aspirin and a form of steroids (methylprednisolone, prednisone, dexamethasone, or steroid pulse) have been used [75]. However, recent systemic reviews [76, 77] and a consensus opinion paper [78] suggested a limited success with retrobulbar hyaluronidase injection. Overall, there is no accepted universal protocol for the use of hyaluronidase in management of vascular complications caused by HA injections. Localised injections of 500–1500 units of hyaluronidase with hourly intervals as soon as vascular occlusion is detected has been suggested [8, 79]. It appears that lower doses (150-300 units), delayed injection, or localised infiltration of hyaluronidase [16, 57] are less effective in management of vascular complications compared to intra-arterial injections of higher doses (1500 units) of hyaluronidase [16]. Future studies are therefore, warranted to establish the ideal injection root (local or intra-arterial), the optimal dose of hyaluronidase, as well as additional treatments (i.e., antibiotics, glucocorticoids, other thrombolytics) for management of skin necrosis and visual impairment complications.

The HA slowly breaks down, binding to more water, maintaining the same volume progressively (isovolumetric degradation), contributing to the delayed occurrence of the oedema years after a successful HA treatment [74, 80], which is the cause of concern for periocular HA injection [61, 62]. Based on 2 case series [61, 62], we know that hyaluronidase maybe more successful in managing periorbital oedema in the upper than the lower eyelid.

There is no universal protocol for reversal of hyaluronic acid fillers and different brand of HA fillers present with various degrees of cross-linkage requiring different amount of hyaluronidase for dissolution [81]. Hyaluronidase also dissolves native hyaluronic acid, but body will restore native HA in 15–20 hours [2]. A limitation of the reviewed studies [52–55] is the use of subjective visual detection [54] or finger palpation using a 5-point scale system [52–55] that may not be able to detect and record subtle changes in palpation scores. Recently, there has been an increase in the use of ultrasound–guided methods [82, 83] and ultrasound can be used in future studies to quantitatively assess the changes in the volume of HA filler over time.

There is a bit of dichotomy of the RTC trial designs and typical use of HA fillers. HA fillers is almost always injected below the skin in real clinical situations and sometimes over the bone. However, studies typically follow intradermal injection depth of HA (intradermal), followed by injection of hyaluronidase intra-lesionally. The failed dissolution of HA filler following multiple hyaluronidase injections, can to be caused by incorrect depth of injection. If fascial structures or muscles lie between the site of HA filler and the site of hyaluronidase hyaluronidase injection, dissolution is dramatically reduced, especially the HA filler exists in a large bolus. Withing this context, the use of ultrasound–guided methods [82, 83] can be a good adjunct to locate the filler and assess quantitatively HA filler degradation after hyaluronidase injection.

The study by Vartanian et. al. [52] lacked a third group without any post-injection to assess the changes post HA injection and compare it to saline and hyaluronidase injections. They recommended a small dose of hyaluronidase equivalent to 5–10 U (0.1–0.2 mL of drug at 50 U/mL) to be injected initially to avoid an allergic reaction, 2 weeks after HA injection. Juhász et. al. [53] also reported that lower concentrations (20 Units) of hyaluronidase were just as effective as higher concentrations (40 Units) in the removal of injected HA filler. Repeated weekly low-volume (0.1 mL) and low-doses of hyaluronidase (9 units) seem to be effective, in particular for correction of minor irregularities [54]. This reduced the risk hypersensitivity and major changes in the affected area.

Another limitation is that the 0.2–0.4 mL of injected HA filler that was used in 4 studies is very small and not representative of the volumes used when aesthetic practitioners use HA filler. These small volumes (0.2–0.4 mL) may be easily cleared by intrinsic enzymes, and therefore future studies may need to study the reversal of larger volumes of injected HA filler. Non-significant differences in the rate of degradation of different HA fillers [52, 53], and different hyaluronidase doses can also be due to the small sample sizes in the 4 studies and future studies may benefit from larger sample sizes.

Alam et. al. [54] introduced the scalping concept with injections of small amount (0.1 mL) of low dose hyaluronidase (1.5–9 units) to achieve the desired effect. A treat to effect concept has therefore been suggested for elective removal of HA fillers with injection intervals of 2 weeks [13]. Heydenrych et. al. [79] recommended an intralesional injections of 4–40 IU per 0.1 mL of injected HA filler [79]. This all bears similarity to the findings of the 5 reviewed RCTs in this systemic review, which was 1.25–37.5 units of hyaluronidase (single injections) per 0.1 mL of injected HA filler. As suggested by Jones et al. [84] injection of 10 units of hyaluronidase for removal of every 0.1 mL of HA filler appears to be a safe starting point and can be repeated every 2 week to achieve the desired effect.

Overall, comparison between the 5 RCTs that assessed the efficacy of hyaluronidase was difficult as they used different formulations of HA filler or hyaluronidase, and HA filler brands may not react similarly to different hyaluronidases [81]. In addition, there were different time intervals and protocols; however, it was evident that hyaluronidase was effective in reversal/removal of uncomplicated HA fillers located at the forearm, upper arm, and back skin. Further studies with larger sample sizes are warranted to establish the ideal treatment protocol/dose for reversal of facial HA injections or management of complications associated with aesthetic HA injection.
